# Calorie intake and patient outcomes in severe acute kidney injury: findings from The Randomized Evaluation of Normal vs. Augmented Level of Replacement Therapy (RENAL) study trial

**DOI:** 10.1186/cc13767

**Published:** 2014-03-14

**Authors:** Rinaldo Bellomo, Alan Cass, Louise Cole, Simon Finfer, Martin Gallagher, Joanne Lee, Serigne Lo, Colin McArthur, Shay McGuinness, John Myburgh, Robyn Norton, Carlos Scheinkestel

**Affiliations:** 1 Department of Intensive Care, Austin Hospital, Studley Rd, Heidelberg, Melbourne, Victoria 3084, Australia; 2 Nephrology Division, The George Institute for International Health, Level 10, King George V Building, Missenden Road, Camperdown, Sydney, New South Wales 2050, Australia; 3 Department of Intensive Care, Nepean Hospital, PO Box 63, Penrith, Sydney, New South Wales 2715, Australia; 4 Department of Intensive Care, Royal North Shore Hospital, Pacific Highway, St Leonards, Sydney, New South Wales 2065, Australia; 5 Division of Nephrology, The George Institute for International Health, Level 10, King George V Building, Missenden Road, Camperdown, Sydney, New South Wales 2050, Australia; 6 Division of Biostatistics, The George Institute for International Health, Level 10, King George V Building, Missenden Road, Camperdown, Sydney, New South Wales 2050, Australia; 7 Department of Critical care Medicine, Auckland City Hospital, Park Rd, Grafton 1142, Auckland, New Zealand; 8 Department of Cardiothoracic and Vascular Intensive Care, Auckland City Hospital, Park Rd, Grafton 1142, Auckland, New Zealand; 9 Department of Intensive Care, St George Hospital, Gray Street, Kogarah, Sydney, New South Wales 2217, Australia; 10 The George Institute for International Health, Level 10, King George V Building, Missenden Road, Camperdown, Sydney, New South Wales 2050, Australia; 11 Department of Intensive Care, Alfred Hospital, Commercial Rd, Prahran, Melbourne, Victoria 3181, Australia

## Abstract

**Introduction:**

Current practice in the delivery of caloric intake (DCI) in patients with severe acute kidney injury (AKI) receiving renal replacement therapy (RRT) is unknown. We aimed to describe calorie administration in patients enrolled in the Randomized Evaluation of Normal vs. Augmented Level of Replacement Therapy (RENAL) study and to assess the association between DCI and clinical outcomes.

**Methods:**

We performed a secondary analysis in 1456 patients from the RENAL trial. We measured the dose and evolution of DCI during treatment and analyzed its association with major clinical outcomes using multivariable logistic regression, Cox proportional hazards models, and time adjusted models.

**Results:**

Overall, mean DCI during treatment in ICU was low at only 10.9 ± 9 Kcal/kg/day for non-survivors and 11 ± 9 Kcal/kg/day for survivors. Among patients with a lower DCI (below the median) 334 of 729 (45.8%) had died at 90-days after randomization compared with 316 of 727 (43.3%) patients with a higher DCI (above the median) (*P* = 0.34). On multivariable logistic regression analysis, mean DCI carried an odds ratio of 0.95 (95% confidence interval (CI): 0.91-1.00; *P* = 0.06) per 100 Kcal increase for 90-day mortality. DCI was not associated with significant differences in renal replacement (RRT) free days, mechanical ventilation free days, ICU free days and hospital free days. These findings remained essentially unaltered after time adjusted analysis and Cox proportional hazards modeling.

**Conclusions:**

In the RENAL study, mean DCI was low. Within the limits of such low caloric intake, greater DCI was not associated with improved clinical outcomes.

**Trial registration:**

ClinicalTrials.gov number, NCT00221013

## Introduction

Achieving an adequate daily calorie intake (DCI) is widely considered beneficial in critically ill patients in general and in particular in patients with acute kidney injury (AKI) [1]. Guidelines recommend the early administration of enteral nutrition whenever possible to achieve an energy intake of 25 to 35 Kcal/day and consideration of parenteral nutrition when enteral nutrition cannot achieve such calorie intake goals [2-4]. However, despite the above guidelines, there is also concern that the administration of energy at such levels in critically ill patients may not be advantageous [5]. Some investigators have shown that low calorie nutrition alone may be sufficient [6] or even desirable [7].

In severe AKI patients who require continuous renal replacement therapy (CRRT), there are very limited data on current practice or on the association between energy intake and patient-centered outcomes. In this setting, all studies are almost 20 years old, single center in design, small in size and with replacement fluid or dialysate fluids rich in glucose and/or lactate [8-11]. Such practices are not relevant to modern CRRT [12-14]. Finally, the impact of CRRT itself on caloric expenditure remains controversial as it may both lead to decreased energy expenditure through cooling; increased loss of energy as patients seek to maintain body temperature in the presence of an extracorporeal circuit, or nutrient loss across the filter [15,16].

The Randomized Evaluation of Normal vs. Augmented Level of Replacement Therapy (RENAL) study [17-20], offers a unique opportunity to explore the association between DCI and outcome because of its size and the availability of detailed DCI data. Accordingly, we conducted a secondary analysis of the RENAL study findings to describe current DCI practice in such patients and study the association between DCI and clinical outcomes.

## Methods

The RENAL study was a multicenter, prospective, randomized trial of two levels of intensity of CRRT in 1,508 critically ill patients with AKI conducted in 35 ICUs in Australia and New Zealand [17,21]. The Human Research Ethics Committees of the University of Sydney and all participating institutions approved the study (Additional file 1 provides a list of the institutional review boards that approved the study). Written informed consent was obtained from patients or their person responsible.

The methodological details of the RENAL study were recently reported [17]. In brief, patients were eligible for enrollment if they were critically ill adults who had AKI, were deemed by the treating clinician to require RRT and fulfilled predefined criteria. Eligible patients were randomly assigned to continuous veno-venous hemodiafiltration (CVVHDF) with effluent flow at 40 ml/Kg/hr (higher intensity) or 25 ml/Kg/hr (lower intensity). Study treatment was discontinued on death, discharge from ICU, or recovery of renal function. The primary study end point was death from any cause by day 90.

### Daily calorie intake

The study did not prescribe any nutritional intake protocol. Nutritional therapy was left to the discretion of attending clinicians. In all patients, DCI was calculated as the sum of all calories administered each day with the exclusion of protein nitrogen. For each patient a mean was calculated during the study period using the DCI value for each day. For the purpose of the study, calorie intake included: a) all glucose given parenterally as part of either drug infusions in 5% glucose or maintenance fluid containing glucose; b) any parenteral nutrition; c) all lipids administered as part of parenteral nutritional solutions, and d) all carbohydrate or lipid-derived calories administered as enteral nutritional solutions. Propofol intake was taken into account. According to the study protocol, DCI data were obtained until the first occurrence of either death, or ICU discharge or the completion of 28 days from study randomization (study treatment period).

### Statistical analysis

Continuous variables were expressed as means with SD for normally distributed variables and as median and IQR for non-normally distributed variables. Comparisons were made using the Student *t*-test or the Mann-Whitney test where appropriate. We divided patients into two groups according to mean DCI calculated for each patient during the study period, low DCI when the individual mean DCI was lower than the median value for the study population and high DCI when individual mean DCI was greater than the median value. Patients with lower and higher DCI were compared by univariate analysis. We then compared the DCI of survivors and non-survivors for DCI and the progressive change over time in DCI. Mean DCI-related variables (dichotomized and continuous) were then assessed for their independent relationship with survival by multivariable logistic regression analysis with adjustment for co-linearity and with adjustment for the following variables: treatment group, acute physiology and chronic health evaluation (APACHE) III score, APACHE III diagnostic groups, daily use of CRRT, age, time from ICU to randomization, presence of sepsis, sequential organ failure assessment (SOFA) respiratory score, SOFA coagulation score, SOFA liver score, SOFA cardiovascular score, SOFA renal score, presence of non-renal organ failure, international normalized ratio (INR) for prothrombin time, activated partial thromboplastin time (APPT), platelet count, serum creatinine, arterial partial pressure of oxygen/inspired oxygen fraction (PaO2/FiO2) ratio, tension of carbon dioxide in arterial blood (PaCO2), pH, glucose, albumin, hemoglobin, use of mechanical ventilation, mean daily fluid balance, and clinical diagnosis of significant edema at randomization.

Multivariable linear regression analysis was similarly used to assess the relationship between individual mean DCI and mechanical ventilation-free days; RRT-free days; ICU-free days and hospital-free days at 90 days, as the dependent variables. Unadjusted analysis of time to death within 90 days of randomization used the Kaplan-Meier product limit estimates and compared survival curves using the log-rank test. To assess whether post-ICU treatment might have affected our findings, we also estimated the relationship between DCI and mortality censored at 28 days or ICU discharge. To test the robustness of any association between mortality and DCI, additional models were applied to data analysis. Such multivariable models included time-adjusted modeling with a cut off of 96 hrs (to exclude patients who died before full nutrition was achieved) and Cox proportional hazards modeling applying the same adjustments for variables included in the logistic regression model. A two-sided *P*-value <0.05 was taken to indicate statistical significance. Statistical analyses were performed and independently checked with the use of SAS software, version 9.1.

## Results

Of the 1,508 patients enrolled in the RENAL study, complete DCI data were available for 1,456 (96.6%). The characteristics of study patients according to whether they received low or high amounts of DCI are compared in Table 1 and are significantly different between the two groups.

**Table 1:** Comparison of baseline characteristics for patient with low (below median) and high (above median) mean daily calorie intake (DCI)

**Baseline characteristics**	**Low DCI, n = 729**	**High DCI, n = 728**	***P*-value**
Age	65.4 (14.8)	64.7 (14.9)	0.022
Male sex	457 (62.7%)	484/728 (66.5%)	0.129
eGFR	53.0 (30.9)	60.1 (30.7)	0.001
Mechanical ventilation	437 (59.9%)	639 (87.8%)	<0.001
Severe sepsis at baseline	307 (42.1%)	412 (56.6%)	<0.001
APACHE III score	103.4 (25.8)	101.5 (25.6)	0.163
SOFA cardiovascular	2.7 (1.6)	3.0 (1.4)	<0.001
SOFA respiration (score)	2.5 (1.1)	3.0 (0.7)	<0.001
SOFA coagulation (score)	0.8 (1.1)	1.1 (1.2)	<0.001
SOFA liver (score)	0.9 (1.2)	1.0 (1.1)	0.300
Weight	79.9 (12.8)	81.4 (13.0)	0.029
Source of admission			
Accident and emergency department	187/686 (27.3%)	161/679 (23.7%)	0.003
Hospital floor/ward	215/686 (31.3%)	172/679 (25.3%)	
Transfer from another ICU	43/686 (6.3%)	66/679 (9.7%)	
Transfer from another hospital	65/686 (9.5%)	89/679 (16.6%)	
Operating room/recovery after emergency surgery	91/686 (13.3%)	113/679 (11.5%)	
Operating room/recovery after elective surgery	85/686 (12.4%)	78/679 (14.1%)	
Non-operative admission diagnosis			
Cardiovascular	245/536 (45.7%)	287/510 (56/3%)	<0.001
Genitourinary	177/536 (33.0%)	52/510 (10.2%)	
Gastrointestinal	39/536 (7.3%)	36/510 (7.1%)	
Hematology	10/536 (1.9%)	12/510 (2.4%)	
Metabolic/endocrine	14/536 (2.6%)	11/510 (2.2%)	
Neurologic	4/536 (0.7%)	6/511 (1.2%)	
Respiratory	43/536 (8.0%)	103/511 (20.2%)	
Transplant	4/536 (0.7%)	1/511 (0.2%)	
Trauma	0/536 (0.0%)	2/511 (0.4%)	
Operative admission diagnosis			
Cardiovascular	131/193 (67.9%)	137/218 (62.8%)	0.256
Genitourinary	3/193 (1.6%)	1/218 (0.5%)	
Gastrointestinal	39/193 (20.2%)	57/218 (26.1%)	
Neurologic	3/193 (1.6%)	4/218 (1.8%)	
Respiratory	3/193 (1.6%)	5/218 (2.3%)	
Transplant	7/193 (3.6%)	2/218 (0.9%)	
Trauma	7/193 (3.6%)	12/218 (5.5%)	
Plasma urea (mmol/L)	23.7 (13.8)	23.3 (11.7)	0.542
Creatinine at randomization (μmol/L)	369 (231)	300 (142)	<0.001
pH	7.2 (0.1)	7.3 (0.1)	<0.001
Bicarbonate (mmol/L)	17.1 (5.8)	19.5 (5.6)	<0.001
Base excess (mEql/L)	−9.7 (6.9)	−6.9 (6.7)	<0.001

Continuous variables expressed as mean with standard deviation in brackets. Nominal variables expressed as number with percentage in brackets. SOFA, sequential organ failure score; RRT, renal replacement therapy; MV, mechanical ventilation; APACHE, acute physiology and chronic health evaluation; eGFR, estimated glomerular filtration rate.

Among patients with a low mean DCI, 334 of 729 (45.8%) had died 90 days after randomization, compared with 316 of 727 (43.3%) patients who received a mean DCI above the median value (*P* = 0.34). Moreover, mean DCI was 867 Kcal/day, with a value among non-survivors of 847 Kcal/day (10.9 Kcal/Kg/day) compared with 883 Kcal/day (11.0 Kcal/Kg/day) among survivors (*P* = 0.32) (Table 2). Mean calorie to protein ratio was 24.9, with a value of among non-survivors of 25.2 compared with 24.7 among survivors (*P* = 0.39).

**Table 2:** Daily calorie intake (DCI) according to survival status at 90 days after randomization

**Baseline characteristics**	**Non-survivors, n = 654**	**Survivors, n = 810**	***P*-value**
Mean DCI during study			
Number	649	807	0.3185
Mean calories (SD)	846.7 (681)	883.3 (709)	
Q1 Q2 Q3	148.0 839.7 1412	90.0 905.8 1447	
Missing	5	3	
Weight-adjusted mean DCI during study			
Number	649	807	0.8086
Mean calories/kg (SD)	10.9 (9.0)	11.0 (9.0)	
Q1 Q2 Q3	1.7 10.4 17.4	1.1 11.2 17.6	
Missing	5	3	

Refers to index admission to a maximum of 28 days (trial treatment); weight-adjusted DCI/patient weight in Kg; Q, quartile.

**Figure 1:**
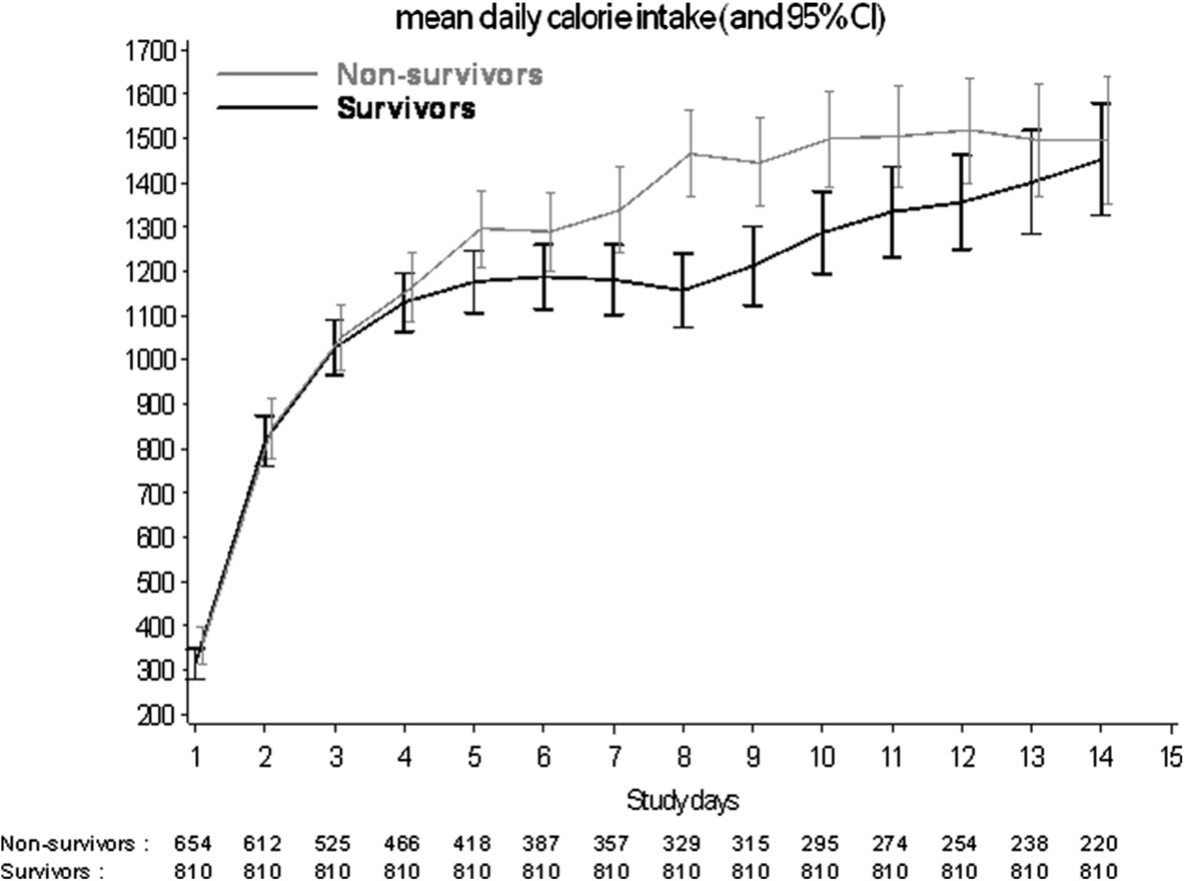
Graphic representation of mean daily caloric intake (DCI) over the first 2 weeks of observation after randomization according to survival status at 90 days.

**Figure 2:**
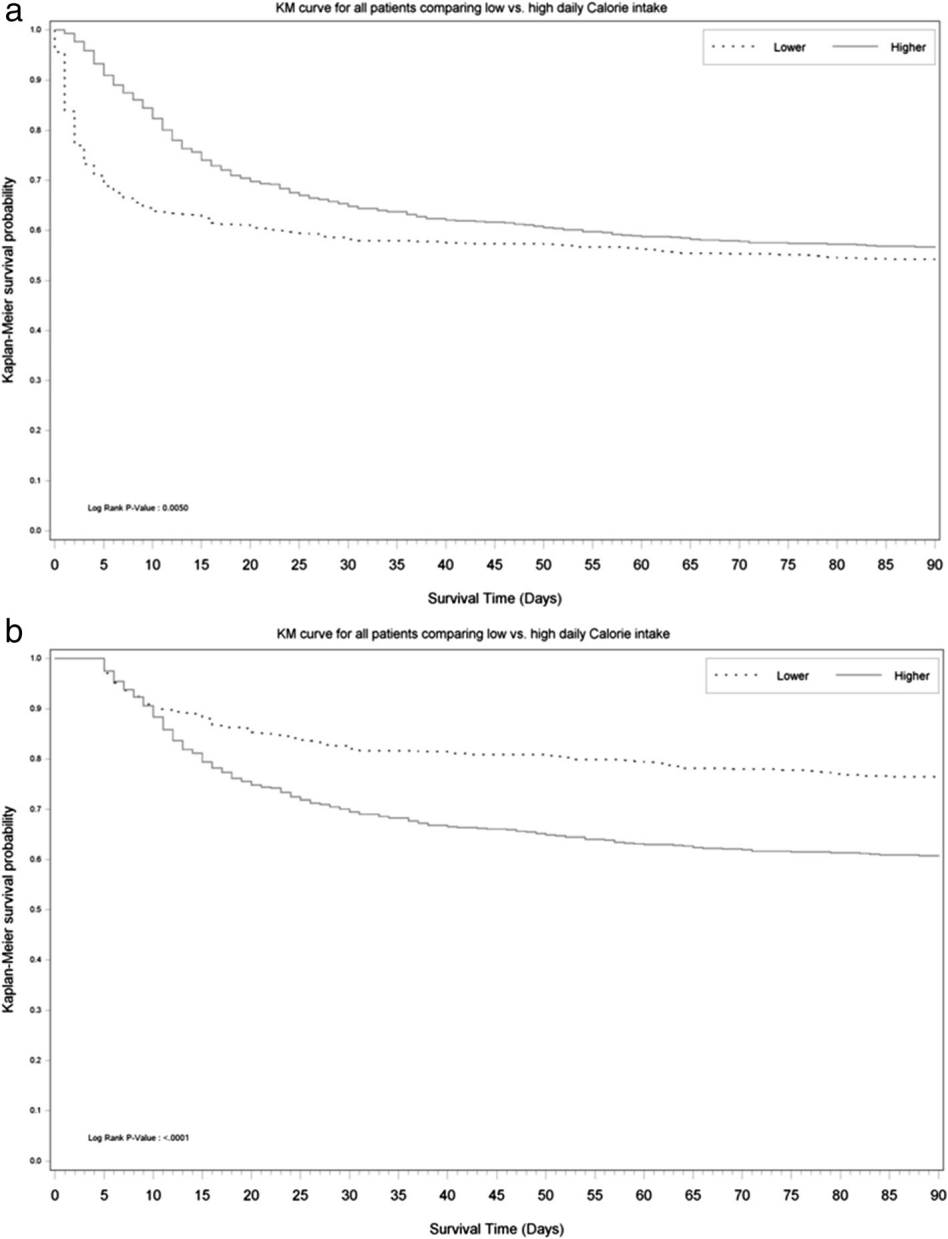
**Kaplan-Meier plots before and after excluding early deaths. (a)** Kaplan-Meier graph of survival plots from randomization to day 90 stratified by the delivery of lower (below median) or higher (above median) daily calorie intake (DCI) during the index ICU admission. No *P*-values are provided as the plot is not adjusted for confounders and is shown to emphasize the reversal of the curve (see Figure 2b) once early deaths are excluded. **(b)** Kaplan-Meier graph of survival plots from randomization to day 90 stratified by the delivery of lower (below median) or higher (above median) DCI during the index ICU admission, after exclusion of patients who died in the first 96 hrs. No *P*-values are provided as the plot is not adjusted for confounders and is shown to emphasize the reversal of the curves (see Figure 2a) once early deaths are excluded.

Overall, 874 patients received enteral nutrition only on 8,334 study days (69.1%), and 382 patients received parenteral nutrition only for a total of 1,667 (13.8%) study days and 200 patients received a combination of enteral and parenteral nutrition for a total of 2,055 (17.1%) study days. The daily DCI for survivors and non-survivors for the first 14 days of observation is displayed in Figure 1. DCI in both groups tended to increase over time reaching a near plateau after approximately 96 hrs. The unadjusted time-to event analysis is shown in Figure 2a for all patients and in Figure 2b after removing patients who died in the first 96 hrs.

On multivariable logistic regression analysis, only a few variables remained independently associated with 90-day mortality (Table 3). Importantly, increased daily DCI during study treatment was not independently associated with decreased mortality. On multivariable linear regression analysis, DCI also showed no association with decreased RRT-free days at day 90 after randomization. When the outcome was survival at 28 days or ICU discharge, there was still no association between DCI above the median value and outcome (odds ratio (OR) 1.02; 95% CI 0.61, 1.71; *P* = 0.93). When a DCI >25 Kcal/Kg/day was used to indicate adequate calorie intake, no significant association was found (OR 0.93; 95% CI 0.47, 1.72; *P* = 0.75). Similar findings were seen when the outcomes of interest were RRT-free days, ICU-free days or hospital-free days (Table 4a, b, c).

**Table 3:** Multivariate logistic regression for 90-day mortality

**Variable name**	**Effect (discrete variable)**	**Odds ratio**	**CI (95%)**	***P*-value**
Median daily calorie intake during ICU admission	High versus low	1.079	0.55 2.13	0.8275
Mean daily calorie intake during ICU admission (per 100 Kcal change)		0.953	0.91 1.00	0.0636
Mean fluid balance (input-output) (litre)		2.016	1.61 2.53	<.0001
Patient's age		1.037	1.03 1.05	<.0001
Patient's weight (Kg)		0.989	0.98 1.00	0.0394
Time from ICU admission to randomization (days)		1.002	1.00 1.00	0.0047
SOFA liver (score)	Failure versus normal	3.384	1.55 7.38	0.0022
International normalized ratio		1.200	1.03 1.39	0.0172
Hemoglobin (g/L)		0.992	0.98 1.00	0.0353
Albumin (g/L)		0.977	0.96 1.00	0.0300
PaCO2 (mm/Hg)		1.016	1.00 1.03	0.0249

Daily calorie intake and all variables with *P* <0.05 presented; APACHE, acute physiology and chronic health evaluation; SOFA, sequential organ failure assessment; PaCO2, tension of carbon dioxide in arterial blood.

**Table 4:** Multivariable linear regression for secondary outcomes*

**Variable name**	**Estimate**	**Standard error**	***P*-value**
**a. Multivariable linear regression for RRT-free days**			
Intercept	−40.32510	33.71933	0.2323
Mean daily calorie intake during ICU admission	0.00189	0.00171	0.2695
Median daily calorie intake during ICU admission	−1.09792	2.61145	0.6744
Positive mean fluid balance	−4.09805	0.87492	<.0001
Treatment	−1.97170	0.85163	0.0210
Time from ICU admission to randomization (in days)	−0.01644	0.00469	0.0005
SOFA liver (score)	−1.63540	0.44579	0.0003
APPT	−0.05581	0.02105	0.0083
pH	8.45062	4.27094	0.0484
**b. Multivariate linear regression of ICU-free days**			
Intercept	−221.76773	281.51940	0.4313
Mean daily calorie intake during ICU admission	0.00468	0.00649	0.4714
Median daily calorie intake during ICU admission	2.89089	8.90361	0.7456
Positive mean fluid balance	−19.69578	3.36383	<.0001
Positive ventilation: no = reference group	−13.45268	5.58339	0.0165
Patient's weight (Kg)	0.45320	0.13896	0.0012
Time from ICU admission to randomization (days)	−0.03736	0.01756	0.0340
Last creatinine concentration	0.11279	0.04434	0.0114
**c. Multivariate linear regression of hospital-free days**			
Intercept	25.05875	258.60573	0.9229
Mean daily calorie intake during ICU admission	−0.00349	0.00601	0.5625
Median daily calorie intake during ICU admission	2.11260	8.32222	0.7997
Positive mean fluid balance	−16.17529	3.08521	<.0001
Patient's weight (Kg)	0.29499	0.12933	0.0231
Last serum urea concentration	−3.72244	1.85441	0.0454
Last creatinine concentration	0.09767	0.04131	0.0185
Glucose (mmol/L)	1.09456	0.50830	0.0319

*Only DCI related variable and significant variables reported. SOFA, sequential organ failure assessment; activated partial thromboplastin time.

The association of DCI with outcomes was also tested by means of additional time-adjusted modeling (1,183 patients were still alive after 96 hrs) and Cox proportional hazards modeling. Both modeling approaches confirmed the main study findings (see Additional file 2).

## Discussion

### Statement of key findings

We used data from a multicenter, randomized, controlled trial of the intensity of CRRT in critically ill patients with AKI to describe current calorie administration practice and to assess the association between DCI and clinical outcomes. We found that overall mean DCI was low at approximately 11 Kcal/Kg/day. In addition, we found that patients with a high DCI (above the median) had similar mortality to patients with a low DCI (below the median). Finally, non-survivors had a similar DCI to survivors. When we estimated the independent association between DCI and outcome at day 90, a high DCI was not independently associated with a significant decrease in the OR for 90-day mortality. To further test the robustness of this finding we performed additional time-adjusted analyses and Cox proportional hazards modeling. These analyses found no independent association between DCI and 90-day mortality or other clinical outcomes.

### Comparison with previous studies

No other studies have reported current calorie delivery practice in patients with AKI. In general critically ill patients however, a recent multicenter observational study in 167 ICU’s found that mean DCI was 14 Kcal/Kg/day [22], a value only slightly above that found in our study. In a recent multicenter trial of intensive insulin therapy in critically ill patients [23], mean DCI was approximately 11 Kcal/Kg/day, a value identical to that delivered to our patients. Thus, current calorie administration practice in Australia and New Zealand (ANZ) is similar to current ICU practice worldwide. In the multicenter observational study of nutrition in general ICU patients cited above, greater mean DCI appeared associated with improved survival. However, no adjustment was made for the competing risk of death [24]. Such bias can be clearly demonstrated in critically ill patients [23,25] where mean DCI increases with time. Thus, patients who die early inevitably receive fewer calories. This pattern creates an artificially inflated chance of an apparent association between greater mean DCI and survival.

Authors [26] and guidelines [3,4,27] continue to recommend a DCI of at least 25 to 35 Kcal/Kg/day in AKI patients, yet, the evidence supporting such recommendations is weak and based on small to very small single-center studies with physiological outcomes only. Moreover, although such recommendations appear reasonable from a physiological and energy expenditure grounds [28-30], no randomized controlled trials (RCTs) exist to compare, for example 10 Kcal/Kg/day (current practice) to 30 Kcal/Kg/day (recommended practice) of energy intake in AKI patients. In support of the need for RCTs, recent investigations have found that permissive underfeeding, trophic feeding or delayed parenteral feeding may be equivalent or perhaps even superior to currently recommended approaches [5-7,25].

### Significance of study findings

These findings from the RENAL study provide the first data on current nutritional practice in patients with severe AKI. They also provide novel information on the relationship between mean DCI and outcome. Such information was collected as part of large multicenter study with independent data verification and negligible missing data. They also provide such information in the setting of essentially exclusive CRRT use. This difference is important because intermittent hemodialysis (IHD) has been shown to limit the ability to control volume status and uremia in critically ill patients with AKI [29,31], thereby potentially impeding full nutritional intake. On the other hand, with CRRT, volume control and full nutritional therapy are free of the limitations imposed by IHD. Thus, given that fluid accumulation is not a problem, mean DCI in this setting can be logically taken to reflect therapeutic choices rather than technical limitations.

Our study demonstrates that mean DCI was well below guideline-based targets in patients receiving CRRT. By assessing, for the first time, its relationship with patient outcomes in the setting of prospective and detailed data collection within a large cohort of patients treated with CRRT, our study also provides evidence that within the range of mean DCI provided in this study, there was no robust independent association between greater mean DCI and favorable outcome, including 90-day mortality and other patient-centered outcomes such as mechanical ventilation, ICU- and hospital-free days. In fact, after early deaths were excluded, patients with a DCI above the median were more likely to die. This surprising finding is possibly due to the confounding effect of time (DCI increases with time and patients who are still in ICU as time goes by have failed to improve and are thus more likely to die) but, nonetheless, highlights the lack of a robust and unchanging relationship with DCI which, if present, may be expected to overcome the effect of confounding.

Our findings may provoke further debate on whether caloric intake is an important determinant of outcome; whether caloric targets as set by current guidelines are justified and whether more restrictive approaches may be acceptable or even desirable. The mechanism responsible for the failure of enhanced nutritional intake to change patient outcome may be complex and may depend on both anabolic resistance [32] and in AKI patients, on the unique changes in protein metabolism seen with this condition [33]. Recent data from randomized controlled trials of nutrition in critically ill patients [5-7,25] also suggest that a more conservative approach to caloric delivery may, at the very least, be safe. Given that severe AKI is relatively common in critically ill patients and given such therapeutic uncertainty, RCTs are urgently needed.

### Study strengths and limitations

This study reports observational findings from a large multicenter randomized controlled study of CRRT for AKI. The data were prospectively collected with specific attention to mean DCI and independently monitored for accuracy, and were free of selection bias. As such, they provide the most comprehensive description of mean DCI during CRRT and of its association with outcome to date. All patients had detailed, prospectively collected outcome data with primary outcome at 90 days. This approach avoided informative censoring of competing events and 90-day mortality is free from ascertainment bias unlike other more subjective primary endpoints (for example, infections) sometimes used in the literature. In addition, all patients had prospectively collected demographic, illness severity and biochemical data at baseline that could be used in multivariable models to adjust for the effect of confounders. The statistical analysis was extensive and involved assessment of the time-bias, a factor that can easily confound the association between nutritional intake and outcome.

On the other hand, the range of DCI was small, thus, despite being the largest study to date, we may have insufficient power to detect an independent association due to the limited number of patients with a DCI >25 Kcal/Kg/day. We could not account for unrecorded variables (such as gastrointestinal dysfunction) that may have affected DCI. We do not have information to explain why caloric intake was low and why it took an average of approximately 4 days for nutrition to reach a plateau. Moreover, data were only available from the time of randomization and did not provide information on mean DCI prior to treatment or after 28 days or ICU discharge. However, the fact that in the RENAL trial the time between ICU admission and randomization was <2 days and the mean duration of study time was approximately 13 days all suggest that the pre-randomization period was unlikely to materially affect the study findings. In addition, the sensitivity analysis showing that the 28-day outcome assessment leads to the same findings as the 90-day outcome assessment provides evidence that interventions after day 28 or ICU discharge are unlikely to have influenced our observations. We did not collect information on the daily dose of propofol infusion. Thus, we cannot quantify its caloric contribution. We do not have information on insulin intake and glucose control. However, glucose management in ANZ has remained steady over the last decade with a mean glucose value of approximately 8 mmol/L [34]. We do not have information on the caloric input derived from normal oral intake. However, such intake was uncommon in these critically ill patients while in ICU and is difficult to quantify. We consider that its overall contribution was negligible. Finally, we do not report on the calories delivered to patients by means of CRRT because its estimate is problematic. All CRRT was performed in all patients with bicarbonate fluids containing 1 g of glucose per liter (5.55 mmol/L) thus potentially delivering 200 to 300 Kcal/day. However, half of the fluid was administered as dialysate, where glucose movement into the patient’s blood stream would be dependent on the glucose gradient and dynamically influenced by the patient’s glucose level. Thus, in hyperglycemic patients, CRRT may have resulted in glucose and caloric loss in hyperglycemic patients and in caloric gain in normoglycemic patients. Such losses and gains would have varied over time according to glycemia, filter function, down time and CRRT intensity making correct estimates essentially impossible.

## Conclusions

In the RENAL study, overall mean DCI was low. However, patients with a lower mean DCI had similar mortality than those with a higher DCI and non-survivors had a similar mean DCI to survivors. After correction for multiple confounding variables and the application of different statistical modeling techniques, a lower mean DCI was not robustly independently associated with increased risk of death at 90 days, or with other major clinical outcomes. Higher-level evidence is needed to better define the optimal DCI target in this important subgroup of patients.

## Key messages

•In the largest multicenter study of AKI treatment with CRRT to date, the average mean DCI was low at 11 KCal/Kg/day

•In severe AKI patients stable calorie intake was only achieved at 4 to 5 days after randomization

•Patients with a low or high mean DCI had similar mortality rates

•Mean DCI was similar among survivors and non-survivors

•After adjustment for multiple confounders, increased daily DCI during study treatment was not independently associated with decreased mortality, decreased RRT-free days ICU-free days or hospital-free days

## Abbreviations

AKI: acute kidney injury; ANZ: Australia and New Zealand; APACHE: acute physiology and chronic health evaluation; APPT: activated partial thromboplastin time; CRRT: continuous renal replacement therapy; CVVHDF: continuous veno-venous hemodiafiltration; DCI: daily calorie intake; FiO2: inspired oxygen fraction; IHD: intermittent hemodialysis; INR: international normalized ratio; OR: odds ratio; PaCO2: tension of carbon dioxide in arterial blood; PaO2: arterial partial pressure of oxygen; RCT: randomized controlled trial; RENAL: Randomized evaluation of normal vs. augmented level of replacement therapy study; RRT: renal replacement therapy; SOFA: sequential organ failure assessment.

## Competing interests

Professor Rinaldo Bellomo reports having received consulting fees as advisor for Gambro.

No other potential conflict of interest relevant to this article was reported.

## Authors’ contributions

RB: conception and design, obtaining of funding to conduct the study; supervision of analysis and interpretation of data; drafting of the manuscript and revising it critically for important intellectual content; writing and final approval of manuscript. AC: conception and design, analysis and interpretation of data; drafting of the manuscript and revising it critically for important intellectual content; obtaining of funding to conduct the study; writing and final approval of manuscript. LC: conception and design, analysis and interpretation of data; drafting of the manuscript and revising it critically for important intellectual content; obtaining of funding to conduct the study; writing and final approval of manuscript. SF: conception and design, analysis and interpretation of data; obtaining of funding to conduct the study, drafting of the manuscript and revising it critically for important intellectual content; writing and final approval of manuscript. MG: conception and design, analysis and interpretation of data; obtaining of funding to conduct the study, drafting of the manuscript and revising it critically for important intellectual content; writing and final approval of manuscript. CM: conception and design, analysis and interpretation of data; obtaining of funding to conduct the study, drafting of the manuscript and revising it critically for important intellectual content; writing and final approval of manuscript. SM: conception and design, analysis and interpretation of data; obtaining of funding to conduct the study, drafting of the manuscript and revising it critically for important intellectual content; writing and final approval of manuscript. JM: conception and design, analysis and interpretation of data; obtaining of funding to conduct the study, drafting of the manuscript and revising it critically for important intellectual content; writing and final approval of manuscript. RN: conception and design, analysis and interpretation of data; obtaining of funding to conduct the study, drafting of the manuscript and revising it critically for important intellectual content; writing and final approval of manuscript. CS: conception and design, analysis and interpretation of data; obtaining of funding to conduct the study, drafting of the manuscript and revising it critically for important intellectual content; writing and final approval of manuscript. JL: acquisition of data, analysis and interpretation of data; drafting of the manuscript and revising it critically for important intellectual content; supervision; writing and final approval of manuscript. SL: acquisition of data, statistical analysis, analysis and interpretation of data; drafting of the manuscript and revising it critically for important intellectual content; writing and final approval of manuscript. All authors meet key authorship requirements and agree to be accountable for all aspects of the work in ensuring that questions related to the accuracy or integrity of any part of the work are appropriately investigated and resolved. All authors read and approved the final manuscript.

## Authors’ information

The RENAL Study Investigators

Australian Capital Territory:

Canberra Hospital: Imogen Mitchell, Rebecca Ashley, Jelena Gissane, Katya Malchukova and Jamie Ranse

New South Wales:

Blacktown Hospital: Asif Raza, Kiran Nand and Treena Sara

Concord Hospital: David Millis, Jeff Tan and Helen Wong

John Hunter Hospital: Peter Harrigan, Elise Crowfoot and Miranda Hardie

Liverpool Hospital: Deepak Bhonagiri and Sharon Micallef

Mater Calvary Hospital, Newcastle: Jorge Brieva and Melissa Lintott

Nepean Hospital: Louise Cole, Rebecca Gresham, Maria Nikas and Leonie Weisbrodt

Prince of Wales Hospital: Yahya Shehabi, Frances Bass, Michelle Campbell and Victoria Stockdale

Royal North Shore Hospital: Simon Finfer, Susan Ankers, Anne O’Connor and Julie Potter

Royal Prince Alfred Hospital: Richard Totaro and Dorrilyn Rajbhandari

St George Hospital: John Myburgh, Vanessa Dhiacou, Alina Jovanovska and Francesca Munster

St Vincent’s Hospital: Priya Nair, Jeff Breeding and Claire Burns

Westmead Hospital: Ashoke Banerjee, Maridy Morrison, Caroline Pfeffercorn and Anne Ritchie

New Zealand:

Auckland City Hospital/CVICU: Shay McGuinness, Heidi Buhr, Michelle Eccleston and Rachael Parke

Auckland City Hospital/DCCM: Colin McArthur, Jeanette Bell and Lynette Newby

Christchurch Hospital: Seton Henderson and Jan Mehrtens

Whangarei Hospital: Michael Kalkoff and Cathy West

Queensland:

Mater Adult and Mater Private Hospital: John Morgan, Lorraine Rudder and Joanne Sutton

Nambour General Hospital: Peter Garrett, Nicole Groves, Shona McDonald, and Jennifer Palmer

Princess Alexandra Hospital: Chris Joyce, Meg Harwood, Jean Helyar and Benjamin Mackie

Royal Brisbane Hospital: Jeff Lipman, Robert Boots, Claire Bertenshaw, Renae Deans, Cheryl Fourie and Melissa Lassig-Smith

South Australia:

Royal Adelaide Hospital: Arthas Flabouris, Jason Edwards, Stephanie O’Connor, and Justine Rivett

Tasmania:

Royal Hobart Hospital: Andrew Turner, Tanya Field and Kathryn Marsden

Victoria:

Austin Hospital: Rinaldo Bellomo, Claire Mathlin, Donna Goldsmith, Inga Mercer and Kim O’Sullivan

Bendigo Hospital: John Edington, Catherine Boschert and Julie Smith

Epworth Hospital: Benno Ihle, Michael Graan and Samuel Ho

Frankston Hospital: John Botha, Nina Fowler, Jodi McInness and Naomi Pratt

Geelong Hospital: Neil Orford, Tania Elderkin, Melissa Fraser and Anne Kinmonth

Monash Medical Centre: Christopher Wright, Sue Burton, Carly Culhane, Pauline Galt and Rebecca Rutzou

Royal Melbourne: Megan Roberston, Deborah Barge, Tania Caf, Belinda Howe and Patzy Low

St Vincent’s Hospital Melbourne: Antony Tobin, Nicole Groves, Jennifer Holmes and Roger Smith

The Alfred Hospital: Carlos Scheinkestel, Andrew Davies, Lynne Murray, Rachael Nevill, Shirley Vallance, Sue Varley and Vickie White

Western Hospital: Craig French, Lorraine Little and Heike Raunow

Western Australia:

Fremantle Hospital: David Blythe and Anna Palermo

Royal Perth Hospital: Geoff Dobb, Melanie Boardman, Jenny Chamberlain, Andree Gould, Geraldine McEntaggart, Samantha Perryman and Linda Thomas

## Supplementary Material

Additional file 1 **Names of ethical bodies that approved the study.** This file contains information on all institutional review boards that have approved the study. Click here for file

Additional file 2: Table S1 Multivariate logistic regression model for day-90 mortality including only patients who survived >96 hrs. **Table S2** Multivariate linear regression of renal replacement therapy (RRT)-free days including only patients who survived >96 hrs. **Table S3** Multivariate linear regression of mechanical ventilation-free days including only patients who survived >96 hrs. **Table S4** Multivariate linear regression of ICU-free days including only patients who survived >96 hrs. **Table S5** Multivariate linear regression of hospital-free days including only patients who survived >96 hrs. **Table S6** Cox regression model for death at day 90. Click here for file
