# Supplementary material for: Calorie intake and patient outcomes in severe acute kidney injury: findings from The Randomized Evaluation of Normal vs. Augmented Level of Replacement Therapy (RENAL) study trial
Source: Crit Care. 2014 Mar 14;18(2):R45. doi: 10.1186/cc13767 (PMC4057152; doi:10.1186/cc13767)
Supplement: Additional file 1 — Names of ethical bodies that approved the study. This file contains information on all institutional review boards that have approved the study. [file cc13767-S1.pdf]

Additional file 1

Title: Names of Ethical Bodies that approved the study

Description: This file contains information on all institutional review boards that have approved the study

List of review boards that approved the study

Human Research Ethics Committee - Canberra Hospital

Human Research Ethics Committee - Blacktown Hospital

Human Research Ethics Committee Concord Hospital

Human Research Ethics Committee - John Hunter Hospital

Human Research Ethics Committee - Liverpool Hospital

Human Research Ethics Committee - Mater Calvary Hospital

Human Research Ethics Committee - Nepean Hospital

Human Research Ethics Committee - Prince of Wales Hospital

Human Research Ethics Committee - Royal North Shore Hospital

Human Research Ethics Committee - Royal Prince Alfred Hospital

Human Research Ethics Committee - St George Hospital

Human Research Ethics Committee - St Vincent's Hospital

Human Research Ethics Committee - Westmead Hospital

Auckland City Hospital Human Research Ethics Committee

Christchurch Hospital Human Research Ethics Committee

Whangarei Hospital Human Research Ethics Committee

Mater Adult and Mater Private Hospital Human Research Ethics Committee

Nambour General Hospital Human Research Ethics Committee

Princess Alexandra Hospital Human Research Ethics Committee:

Royal Brisbane Hospital Human Research Ethics Committee

Royal Adelaide Hospital Human Research Ethics Committee

Royal Hobart Hospital Human Research Ethics Committee

Austin Hospital Human Research Ethics Committee

Bendigo Hospital Human Research Ethics Committee

Epworth Hospital Human Research Ethics Committee

Frankston Hospital Human Research Ethics Committee

Geelong Hospital Human Research Ethics Committee

Monash Medical Centre Human Research Ethics Committee

Royal Melbourne Human Research Ethics Committee

St Vincent's Hospital Melbourne Human Research Ethics Committee

The Alfred Hospital Human Research Ethics Committee

Western Hospital Human Research Ethics Committee

Fremantle Hospital Human Research Ethics Committee

Royal Perth Hospital Human Research Ethics Committee
