# Supplementary material for: Calorie intake and patient outcomes in severe acute kidney injury: findings from The Randomized Evaluation of Normal vs. Augmented Level of Replacement Therapy (RENAL) study trial
Source: Crit Care. 2014 Mar 14;18(2):R45. doi: 10.1186/cc13767 (PMC4057152; doi:10.1186/cc13767)
Supplement: Additional file 2: Table S1 — Multivariate logistic regression model for day-90 mortality including only patients who survived >96 hrs. Table S2 Multivariate linear regression of renal replacement therapy (RRT)-free days including only patients who survived >96 hrs. Table S3 Multivariate linear regression of mechanical ventilation-free days including only patients who survived >96 hrs. Table S4 Multivariate linear regression of ICU-free days including only patients who survived >96 hrs. Table S5 Multivariate linear regression of hospital-free days including only patients who survived >96 hrs. Table S6 Cox regression model for death at day 90. [file cc13767-S2.doc]

**Appendix**

**Table 1: Multivariate Logistic regression model for Day 90 mortality including only patients who survived > 96 hours**

| Variable name | Effect (discrete variable) | Odds ratio | CI(95%) | P-values |
| --- | --- | --- | --- | --- |
| Intercept |  | . |  | 0.3337 |
| Median daily calorie intake during ICU admission | Yes vs No | 1.363 | 0.63 2.93 | 0.4287 |
| Mean daily calorie intake during ICU admission (per 100 Kcal increase) |  | 0.966 | 0.91 1.02 | 0.2086 |
| Mean fluid balance (input-output) (l) |  | 1.618 | 1.23 2.14 | 0.0007 |
| Positive mean fluid balance | Yes vs No | 1.027 | 0.65 1.62 | 0.9096 |
| Treatment: Higher Intensity vs Lower Intensity | Lower vs Higher | 1.092 | 0.81 1.47 | 0.5624 |
| Patients Age |  | 1.042 | 1.03 1.05 | <.0001 |
| Patients Weight (in Kg) |  | 0.989 | 0.98 1.00 | 0.0851 |
| Time from ICU admission to Randomization (in days) |  | 1.002 | 1.00 1.00 | 0.0196 |
| Severe sepsis at baseline | Yes vs No | 1.248 | 0.91 1.72 | 0.1747 |
| SOFA Respiration (score) | Dysfunction vs Normal | 0.818 | 0.30 2.22 | 0.6934 |
|  | Failure vs Normal | 1.949 | 0.32 12.0 | 0.4723 |
| SOFA Coagulation (score) | Dysfunction vs Normal | 1.017 | 0.61 1.70 | 0.9479 |
|  | Failure vs Normal | 1.208 | 0.50 2.89 | 0.6717 |
| SOFA Liver (score) | Dysfunction vs Normal | 0.828 | 0.54 1.28 | 0.3945 |
|  | Failure vs Normal | 3.273 | 1.40 7.66 | 0.0063 |
| SOFA Cardiovascular (score) | Dysfunction vs Normal | 0.688 | 0.37 1.27 | 0.2320 |
|  | Failure vs Normal | 0.542 | 0.24 1.24 | 0.1481 |
| SOFA Renal (score) | Dysfunction vs Normal | 0.871 | 0.26 2.88 | 0.8206 |
|  | Failure vs Normal | 0.736 | 0.20 2.71 | 0.6450 |
| Overall SOFA score (all non-missing organ scores lumped together/5) |  | 1.196 | 0.49 2.94 | 0.6962 |
| Patient had at least 1 non-renal failure failure (SOFA score 3-4) (Y/N) | Yes vs No | 1.139 | 0.54 2.43 | 0.7351 |
| Last creatinine concentration |  | 1.001 | 1.00 1.00 | 0.4570 |
| INR |  | 1.217 | 1.05 1.42 | 0.0107 |
| Haemoglobin (g/L) |  | 0.991 | 0.98 1.00 | 0.0403 |
| Platelet count(x109/L) |  | 0.998 | 1.00 1.00 | 0.0976 |
| Sodium (mmol/L) |  | 0.992 | 0.97 1.02 | 0.5378 |
| Creatinine (µmol/L) |  | 0.998 | 1.00 1.00 | 0.3044 |
| Albumin (g/L) |  | 0.979 | 0.96 1.00 | 0.0706 |
| Magnesium (mmol/L) |  | 1.110 | 0.72 1.71 | 0.6340 |
| pH |  | 1.963 | 0.42 9.19 | 0.3916 |
| PaCP2 (mm/Hg) |  | 1.014 | 1.00 1.03 | 0.0682 |
| Mechanical ventilation | Yes vs No | 0.597 | 0.08 4.23 | 0.6060 |

T:\Statistics\Projects\RENAL\Final analysis\Exploratory Analysis\Calorie Analysis\Programs\a_logistic_excludingDeath_D5.sas

Data cutoff: 16JAN2009 Last run: 14DEC2012 9:21

**Table 2: Multivariate Linear regression of ''RRT-free days'' including only patients who survived > 96 hours.**

| **Variable name** | **Estimates** | **Std Error** | **P-values** |
| --- | --- | --- | --- |
| Intercept | -12.69807 | 32.54974 | 0.6967 |
| Mean daily calorie intake during ICU admission | -0.00088943 | 0.00157 | 0.5715 |
| Median daily calorie intake during ICU admission | -3.76103 | 2.53564 | 0.1388 |
| Positive mean fluid balance | -0.87982 | 0.82844 | 0.2889 |
| TREAT | -0.83311 | 0.79015 | 0.2923 |
| Fluid overload at randomization | 0.57225 | 0.81062 | 0.4806 |
| Time from ICU admission to Randomization (in days) | -0.01058 | 0.00462 | 0.0224 |
| SEPSIS: No== reference grp | 0.86536 | 0.86304 | 0.3166 |
| Apache III score | -0.00816 | 0.01847 | 0.6591 |
| SOFA Respiration (score) | 0.64866 | 0.68989 | 0.3477 |
| SOFA Coagulation (score) | 0.87443 | 0.53134 | 0.1006 |
| SOFA Liver (score) | -1.16260 | 0.42604 | 0.0066 |
| SOFA Cardiovascular (score) | 0.07919 | 0.33277 | 0.8120 |
| SOFA Renal (score) | -0.70119 | 0.51212 | 0.1717 |
| Patient had at least 1 non-renal failure failure (SOFA score 3-4) (Y/N) | -1.25644 | 1.73275 | 0.4688 |
| Last Serum urea concentration | 0.27584 | 0.32532 | 0.3970 |
| Last creatinine concentration | 0.00302 | 0.00656 | 0.6462 |
| INR | 0.03054 | 0.39206 | 0.9380 |
| APPT | -0.05578 | 0.02210 | 0.0120 |
| Platelet count(x109/L) | 0.00956 | 0.00464 | 0.0400 |
| Sodium (mmol/L) | 0.04859 | 0.06715 | 0.4698 |
| Urea (mmol/L) | -0.32877 | 0.32671 | 0.3149 |
| Creatinine (µmol/L) | 0.00036753 | 0.00595 | 0.9508 |
| Albumin (g/L) | 0.09948 | 0.06059 | 0.1014 |
| Magnesium (mmol/L) | -1.49291 | 1.09554 | 0.1737 |
| pH | 3.92572 | 4.17647 | 0.3478 |
| PaCP2 (mm/Hg) | -0.01677 | 0.04097 | 0.6826 |
| Positive Ventilation: No==refrence grp | -1.56676 | 1.59539 | 0.3267 |
| Glomerular filtration rate (eGFR) | 0.04499 | 0.02306 | 0.0517 |
| Glomerular filtration rate (eGFR) < 45ml/min: No==ref grp | 3.47226 | 1.53906 | 0.0246 |
| Glomerular filtration rate (eGFR) > 60 ml/min: No==ref grp | 0 | . | . |

T:\Statistics\Projects\RENAL\Final analysis\Exploratory Analysis\Calorie Analysis\Programs\a_logistic_excludingDeath_D5.sas

Data cutoff: 16JAN2009 Last run: 14DEC2012 9:21

**Table 3 : Multivariate Linear regression of ''MECHANICAL VENTILATION-free days'' including only patients who survived > 96 hours**

| Variable name | Estimates | Std Error | P-values |
| --- | --- | --- | --- |
| Intercept | 619.40873 | 359.42629 | 0.0858 |
| Mean daily calorie intake during ICU admission | -0.00122 | 0.00669 | 0.8551 |
| Median daily calorie intake during ICU admission | -5.82650 | 9.93636 | 0.5580 |
| Positive mean fluid balance | -9.72105 | 3.53170 | 0.0062 |
| TREAT | 3.14821 | 3.48283 | 0.3667 |
| Fluid overload at randomization | -1.19994 | 3.58995 | 0.7384 |
| Patients Age | -0.12638 | 0.13980 | 0.3667 |
| Sex: Male==reference grp | 1.86718 | 3.76855 | 0.6206 |
| SEPSIS: No== reference grp | -0.59023 | 3.92629 | 0.8806 |
| Patient had at least 1 non-renal failure failure (SOFA score 3-4) (Y/N) | 6.22460 | 7.64226 | 0.4159 |
| Positive Ventilation: No==refrence grp | -9.93773 | 5.81766 | 0.0885 |
| Glomerular filtration rate (eGFR) > 60 ml/min: No==ref grp | 4.84529 | 6.57859 | 0.4619 |
| Patients Weight (in Kg) | 0.43183 | 0.14320 | 0.0028 |
| Apache III score | -0.17382 | 0.07925 | 0.0290 |
| Overall SOFA score (all non-missing organ scores lumped together/5) | -1.16671 | 4.54196 | 0.7974 |
| Last Serum urea concentration | -2.69559 | 1.97393 | 0.1730 |
| Last creatinine concentration | 0.05317 | 0.04750 | 0.2639 |
| Haemoglobin (g/L) | 0.13374 | 0.09719 | 0.1697 |
| White cell count (x109/L) | 0.12196 | 0.12245 | 0.3200 |
| Platelet count(x109/L) | -0.00103 | 0.01627 | 0.9495 |
| Sodium (mmol/L) | 0.23679 | 0.49113 | 0.6300 |
| POTASSIUM (mmol/L) | 0.88533 | 2.34293 | 0.7058 |
| Chloride (mmol/L) | 0.33119 | 0.49034 | 0.4999 |
| Bicarbonate (mmol/L) | 0.55163 | 0.78387 | 0.4821 |
| Urea (mmol/L) | 2.12247 | 1.97579 | 0.2835 |
| Creatinine (µmol/L) | -0.02843 | 0.04631 | 0.5397 |
| Phosphate (mmol/L) | -2.36262 | 3.23805 | 0.4661 |
| Albumin (g/L) | 0.11315 | 0.26235 | 0.6665 |
| Magnesium (mmol/L) | -0.76930 | 4.96695 | 0.8770 |
| pH | -82.36619 | 45.76094 | 0.0728 |
| PaCP2 (mm/Hg) | -0.92903 | 0.35572 | 0.0094 |
| Base excess (mmol/L) | 1.14449 | 0.81695 | 0.1622 |
| iCa++ (mmol/L) | -6.29264 | 9.28033 | 0.4982 |
| Glucose (mmol/L) | 1.04625 | 0.57259 | 0.0686 |
| Glomerular filtration rate (eGFR) | 0.03350 | 0.10124 | 0.7409 |

T:\Statistics\Projects\RENAL\Final analysis\Exploratory Analysis\Calorie Analysis\Programs\a_logistic_excludingDeath_D5.sas

Data cutoff: 16JAN2009 Last run: 14DEC2012 9:21

**Table 4 : Multivariate Linear regression of ''ICU-free days'' including only patients who survived > 96 hours**

| Variable name | Estimates | Std Error | P-values |
| --- | --- | --- | --- |
| Intercept | 235.61385 | 365.05573 | 0.5191 |
| Mean daily calorie intake during ICU admission | -0.00102 | 0.00666 | 0.8787 |
| Median daily calorie intake during ICU admission | -1.33435 | 9.34139 | 0.8865 |
| Positive mean fluid balance | -9.56817 | 3.46110 | 0.0060 |
| Fluid overload at randomization | -0.55937 | 3.50714 | 0.8734 |
| Patients Age | 0.15912 | 0.13386 | 0.2355 |
| Sex: Male==reference grp | 2.23709 | 3.68354 | 0.5441 |
| SEPSIS: No== reference grp | 0.48091 | 3.77389 | 0.8987 |
| Patient had at least 1 non-renal failure failure (SOFA score 3-4) (Y/N) | -3.22023 | 7.56995 | 0.6708 |
| Positive Ventilation: No==refrence grp | -8.78152 | 5.64162 | 0.1206 |
| Glomerular filtration rate (eGFR) > 60 ml/min: No==ref grp | 10.70134 | 6.29846 | 0.0903 |
| Patients Weight (in Kg) | 0.39749 | 0.13960 | 0.0047 |
| Time from ICU admission to Randomization (in days) | -0.03691 | 0.01784 | 0.0394 |
| Apache III score | -0.12776 | 0.07644 | 0.0957 |
| Overall SOFA score (all non-missing organ scores lumped together/5) | -1.02041 | 4.37768 | 0.8158 |
| Last Serum urea concentration | -3.01390 | 1.85440 | 0.1051 |
| Last creatinine concentration | 0.07029 | 0.04585 | 0.1263 |
| Haemoglobin (g/L) | 0.13020 | 0.09359 | 0.1652 |
| White cell count (x109/L) | 0.11411 | 0.11636 | 0.3275 |
| Platelet count(x109/L) | 0.01136 | 0.01646 | 0.4907 |
| Sodium (mmol/L) | -0.16230 | 0.47248 | 0.7315 |
| POTASSIUM (mmol/L) | -1.87126 | 2.31619 | 0.4198 |
| Chloride (mmol/L) | 0.44698 | 0.47259 | 0.3450 |
| Bicarbonate (mmol/L) | 0.57277 | 0.75479 | 0.4485 |
| Urea (mmol/L) | 2.57743 | 1.85730 | 0.1662 |
| Creatinine (µmol/L) | -0.03310 | 0.04473 | 0.4599 |
| Phosphate (mmol/L) | -1.58561 | 3.11286 | 0.6109 |
| Albumin (g/L) | 0.01994 | 0.25595 | 0.9380 |
| Magnesium (mmol/L) | -2.61387 | 4.70999 | 0.5793 |
| pH | -29.89063 | 46.42521 | 0.5202 |
| PaCP2 (mm/Hg) | -0.79611 | 0.34870 | 0.0231 |
| Base excess (mmol/L) | 0.19817 | 0.85347 | 0.8165 |
| iCa++ (mmol/L) | 9.70226 | 9.17093 | 0.2909 |
| Glucose (mmol/L) | 0.84532 | 0.55002 | 0.1254 |
| Glomerular filtration rate (eGFR) | 0.15080 | 0.09586 | 0.1168 |

T:\Statistics\Projects\RENAL\Final analysis\Exploratory Analysis\Calorie Analysis\Programs\a_logistic_excludingDeath_D5.sas

Data cutoff: 16JAN2009 Last run: 14DEC2012 9:21

**Table 5 : Multivariate Linear regression of ''HOSPITAL-free days'' including only patients who survived > 96 hours**

| Variable name | Estimates | Std Error | P-values |
| --- | --- | --- | --- |
| Intercept | 676.00663 | 356.28621 | 0.0587 |
| Mean daily calorie intake during ICU admission | -0.01170 | 0.00667 | 0.0806 |
| Median daily calorie intake during ICU admission | 2.58757 | 9.46085 | 0.7846 |
| Positive mean fluid balance | -9.08412 | 3.44357 | 0.0088 |
| Fluid overload at randomization | -2.10040 | 3.47496 | 0.5460 |
| Patients Age | -0.04987 | 0.13659 | 0.7153 |
| Sex: Male==reference grp | 0.89355 | 3.62334 | 0.8054 |
| SEPSIS: No== reference grp | -0.84302 | 3.78242 | 0.8238 |
| Patient had at least 1 non-renal failure failure (SOFA score 3-4) (Y/N) | 8.04283 | 7.28528 | 0.2704 |
| Positive Ventilation: No==refrence grp | -3.34307 | 5.61659 | 0.5521 |
| Glomerular filtration rate (eGFR) > 60 ml/min: No==ref grp | 4.41542 | 6.29179 | 0.4833 |
| Patients Weight (in Kg) | 0.20428 | 0.14116 | 0.1489 |
| Time from ICU admission to Randomization (in days) | -0.01626 | 0.01821 | 0.3728 |
| Apache III score | -0.16457 | 0.07907 | 0.0382 |
| Overall SOFA score (all non-missing organ scores lumped together/5) | -1.35118 | 4.36758 | 0.7572 |
| Last Serum urea concentration | -3.45185 | 1.86445 | 0.0650 |
| Last creatinine concentration | 0.04244 | 0.04567 | 0.3535 |
| Haemoglobin (g/L) | 0.16714 | 0.09598 | 0.0826 |
| White cell count (x109/L) | 0.15996 | 0.11639 | 0.1703 |
| Platelet count(x109/L) | -0.00805 | 0.01590 | 0.6132 |
| Sodium (mmol/L) | 0.14305 | 0.46848 | 0.7603 |
| POTASSIUM (mmol/L) | 0.80113 | 2.26132 | 0.7234 |
| Chloride (mmol/L) | 0.27137 | 0.46856 | 0.5629 |
| Bicarbonate (mmol/L) | 0.30445 | 0.77386 | 0.6943 |
| Urea (mmol/L) | 3.03526 | 1.86902 | 0.1054 |
| Creatinine (µmol/L) | -0.01490 | 0.04439 | 0.7372 |
| Phosphate (mmol/L) | -3.07969 | 3.15389 | 0.3296 |
| Albumin (g/L) | 0.30159 | 0.25442 | 0.2368 |
| Magnesium (mmol/L) | -1.72557 | 4.70355 | 0.7140 |
| pH | -91.43930 | 45.22997 | 0.0441 |
| PaCO2 (mm/Hg) | -0.72363 | 0.34729 | 0.0380 |
| Base excess (mmol/L) | 1.17666 | 0.82585 | 0.1552 |
| iCa++ (mmol/L) | -1.51640 | 8.73942 | 0.8624 |
| Glucose (mmol/L) | 1.19938 | 0.55582 | 0.0317 |
| Glomerular filtration rate (eGFR) | 0.07249 | 0.09665 | 0.4538 |

**Table 6: Cox model regression model for Death* at Day 90**

|  | | Adjusted*** | | |
| --- | --- | --- | --- | --- |
| Variable | Category | Hazard ratio | 95% CI | P-value |
| Median daily calorie intake during ICU admission | Upper vs Lower | 1.154 | (0.631 2.109) | 0.6425 |
| Mean daily calorie intake during ICU admission (per 100 Kcal increase) |  | 0.982 | (0.945 1.022) | 0.3751 |
| Mean fluid balance (input-output) (l) |  | 1.494 | (1.217 1.835) | 0.0001 |
| Positive mean fluid balance | Yes vs No | 0.987 | (0.707 1.377) | 0.9373 |
| Patients Age |  | 1.031 | (1.021 1.040) | <.0001 |
| Patients Weight (in Kg) |  | 0.993 | (0.984 1.002) | 0.1425 |
| Time from ICU admission to Randomization (in days) |  | 1.001 | (1.000 1.001) | 0.0082 |
| Severe sepsis at baseline | Yes vs No | 1.234 | (0.965 1.579) | 0.0938 |
| SOFA Respiration (score) | Dysfunction vs Normal | 0.833 | (0.371 1.872) | 0.6589 |
| SOFA Respiration (score) | Failure vs Normal | 1.378 | (0.317 5.994) | 0.6688 |
| SOFA Coagulation (score) | Dysfunction vs Normal | 1.002 | (0.676 1.484) | 0.9925 |
| SOFA Coagulation (score) | Failure vs Normal | 1.019 | (0.537 1.935) | 0.9532 |
| SOFA Liver (score) | Dysfunction vs Normal | 0.863 | (0.620 1.200) | 0.3805 |
| SOFA Liver (score) | Failure vs Normal | 2.348 | (1.287 4.285) | 0.0054 |
| SOFA Cardiovascular (score) | Dysfunction vs Normal | 0.722 | (0.460 1.134) | 0.1572 |
| SOFA Cardiovascular (score) | Failure vs Normal | 0.594 | (0.323 1.091) | 0.0928 |
| SOFA Renal (score) | Dysfunction vs Normal | 1.147 | (0.475 2.770) | 0.7601 |
| SOFA Renal (score) | Failure vs Normal | 1.014 | (0.384 2.678) | 0.9769 |
| Overall SOFA score (all non-missing organ scores lumped together/5) |  | 1.264 | (0.651 2.457) | 0.4891 |
| Patient had at least 1 non-renal failure failure (SOFA score 3-4) (Y/N) | Yes vs No | 1.124 | (0.618 2.043) | 0.7017 |
| Last creatinine concentration |  | 1.001 | (0.999 1.003) | 0.3966 |
| INR |  | 1.131 | (1.030 1.242) | 0.0098 |
| Haemoglobin (g/L) |  | 0.993 | (0.986 1.000) | 0.0476 |
| Platelet count(x109/L) |  | 0.999 | (0.997 1.000) | 0.1243 |
| Sodium (mmol/L) |  | 0.997 | (0.979 1.017) | 0.7939 |
| Creatinine (µmol/L) |  | 0.999 | (0.996 1.001) | 0.1794 |
| Albumin (g/L) |  | 0.983 | (0.966 1.000) | 0.0538 |
| Magnesium (mmol/L) |  | 1.233 | (0.901 1.688) | 0.1899 |
| pH |  | 1.941 | (0.627 6.015) | 0.2503 |
| PaCP2 (mm/Hg) |  | 1.012 | (1.001 1.024) | 0.0340 |
| Mechanical ventilation | Yes vs No | 0.745 | (0.152 3.650) | 0.7166 |

* Only patients who survived > 96 hrs are considered

** model stratified by treatment allocation (intensive or conventional)

*** Covariates for adjusted model include Calorie intake, Mean daily fluid balance, Positive vs. negative fluid balance Edema, baseline characteristics including patients' age, sex, sepsis (yes or no), organ failure (a respiratory, coagulation, liver, cardiovascular or renal SOFA score),pre-randomization blood phosphate concentration dichotomized at the median value, INR, ALBUMIN, Platelet count, PH and Ventilation (Yes vs. No)
